# Responses to water stress extremes in diverse red clover germplasm accessions

**DOI:** 10.3389/fpls.2023.1195058

**Published:** 2023-06-22

**Authors:** Angus D. Heslop, Zulfi Jahufer, Rainer W. Hofmann

**Affiliations:** ^1^ Faculty of Agriculture and Life Sciences, Lincoln University, Lincoln, New Zealand; ^2^ AgResearch Limited, Lincoln Research Centre, Christchurch, New Zealand; ^3^ AgResearch Limited, Lincoln Research Centre, Palmerston North, New Zealand

**Keywords:** red clover, white clover, water deficit, waterlogging, morphological traits, physiological traits, germplasm

## Abstract

Red clover (*Trifolium pratense* L.), a key perennial pastoral species used globally, can strengthen pastural mixes to withstand increasingly disruptive weather patterns from climate change. Breeding selections can be refined for this purpose by obtaining an in-depth understanding of key functional traits. A replicated randomized complete block glasshouse pot trial was used to observe trait responses critical to plant performance under control (15% VMC), water deficit (5% VMC) and waterlogged conditions (50% VMC) in seven red clover populations and compared against white clover. Twelve morphological and physiological traits were identified as key contributors to the different plant coping mechanisms displayed. Under water deficit, the levels of all aboveground morphological traits decreased, highlighted by a 41% decrease in total dry matter and 50% decreases in both leaf number and leaf thickness compared to the control treatment. An increase in root to shoot ratio indicated a shift to prioritizing root maintenance by sacrificing shoot growth, a trait attributed to plant water deficit tolerance. Under waterlogging, a reduction in photosynthetic activity among red clover populations reduced several morphological traits including a 30% decrease in root dry mass and total dry matter, and a 34% decrease in leaf number. The importance of root morphology for waterlogging was highlighted with low performance of red clover: there was an 83% decrease in root dry mass compared to white clover which was able to maintain root dry mass and therefore plant performance. This study highlights the importance of germplasm evaluation across water stress extremes to identify traits for future breeding programs.

## 1 Introduction

Even with advancement in technology and cultivar development, global agricultural productivity has been reduced by 21% over the last 60 years from changes in precipitation patterns caused by climate change (Ortiz-Bobea et al., 2021). Current trends suggest the continuation of disrupted weather patterns and an increase in frequency of soil waterlogging, caused by flooding, and water deficit events (Siddique et al., 2013). Soil waterlogging occurs when there is an excess of water filling the soil pores, usually around 20% higher than the field capacity (Simova-Stoilova et al., 2012). Approximately 10-12% of the global agricultural land is affected by waterlogging (Kaur et al., 2020). Initially waterlogging restricts gas exchange between atmosphere and soil and reduces the amount of available oxygen, resulting in hypoxia in plant roots (Simova-Stoilova et al., 2012). This reduces photosynthesis and the uptake of nutrients essential for plant growth. The chemical makeup of soil also changes with prolonged waterlogging with a switch from an aerobic to anaerobic conditions (Simova-Stoilova et al., 2012). This results in a build-up of toxic compounds such as sulfides and lactic acid, the consumption of stored carbohydrates and eventually anoxia in the plant roots (Fiedler et al., 2007). Prolonged waterlogging causes adverse effects on plant growth and functioning that can induce early senescence of leaves and plant death (Simova-Stoilova et al., 2012).

Drought is declared when there is a prolonged period of abnormally low rainfall accompanied by high temperatures leading to a shortage of available water (Mir et al., 2012). The impact of drought on crops depends on its intensity, timing, duration, crop species and growth stage of the plant (da Silva et al., 2011; Mir et al., 2012). Under water deficit caused by drought, several physiological and biochemical processes are altered including transpiration, photosynthesis and the metabolism of carbohydrates, amino acids, and other organic compounds (Šircelj et al., 2005). A reduction in soil moisture limits the availability of key nutrients from the soil due to the decreased rate of nutrient diffusion to the absorbing root surface (Sadras and Milroy, 1996). The availability is further reduced due to stomatal closure (da Silva et al., 2011). This reduces transpiration, the process of water movement through a plant which is responsible for the transfer of nutrients from roots to shoots (Bohnert and Jenson, 1996). The development, growth and functioning of key physiological processes is adversely affected under prolonged water deficit, this can induce early leaf senescence and plant death (Bohnert and Jenson, 1996; da Silva et al., 2011).

Water stress is defined as an imbalance between an over or under availability of water and the amount of water needed by the plant, this can occur gradually or suddenly Shaw and Laing, 1966; Kar, 2011). Plant response to water stress varies depending on the speed or occurrence and severity (Kar, 2011). Selecting for tolerance to water stress in plants is complex. It involves multiple quantitative traits each controlled by many genes, along with interactions between plant structure, function, and the environment (Barnabás et al., 2008; Mir et al., 2012). It also depends on the breeder’s interpretation of tolerance that they are selecting for. For farmers this will dictate what crops are grown or what farming management system is implemented. Tolerance of plants to moisture stress can be expressed differently *via* either isohydric (“water saver”) or anisohydric (“water spender”) coping strategies (Domec et al., 2017).

The water spender strategy involves the plant maintaining a higher plant growth rate by using stored carbohydrate reserves. This strategy relies on the plant being able to replenish these carbohydrate stocks post stress. A water spender may switch to reproductive growth prior to severe stress by reducing the length of its lifecycle, using stored carbohydrate reserves to promote a higher rate of growth and by successfully producing seed (Barnabás et al., 2008; Domec et al., 2017). The water saver strategy involves the maintenance of plant function at limited water availability by the senescence of older leaves, reduction in size or adaption of key plant organs and osmotic adjustment (Barnabás et al., 2008; Domec et al., 2017). When developing new cultivars, ensuring genetically diverse backgrounds will increase the ability to tolerate differing environmental stresses by switching between coping strategies depending on stresses as has been shown in a range of species (Domec et al., 2017). A way to do this is through the introduction of raw germplasm material (Egan et al., 2021).

Red clover is a key temperate perennial pastoral legume species used globally. It is recognized as one of the most important legumes in the world, capable of producing forage high in protein and digestibility. It is known for its ability to establish rapidly and grow in a wide range of soil types, pH levels and environmental conditions (Taylor and Quesenberry, 1996). As a legume, red clover can biologically fix nitrogen through forming symbiosis with *Rhizobium*. Red clover tends to have higher summer production compared to other legume species such as white clover due to its extensive taproot system (Taylor and Quesenberry, 1996; Black et al., 2009). Unlike its well-known drought tolerant feature, limited understanding of its physiological and morphological traits responses under waterlogging conditions. From published studies, red clover showed low tolerance to waterlogging with decreases in chlorophyll content and dry matter yield and increases in levels of oxidant stress in leaves (Simova-Stoilova et al., 2012; Stevenson et al., 2017). Other studies reported changes in root chemical composition and protein changes in leaf tissue when red clover populations were under waterlogging stress (Whitehead et al., 1979; Stoychev et al., 2013).

Research has largely focused on nitrogen fixation and nodule morphological changes for other legume species in response to waterlogging (Pugh et al., 1995). Higher tolerance to waterlogging has been observed in white clover (*Trifolium repens* L.) compared to red clover (Huber et al., 2009; Simova-Stoilova et al., 2012). Similarly, work in annual clover species such as subterranean clover (*Trifolium subterraneum* L.), cluster clover (*T. glomeratum* L.), woolly clover (*Trifolium tomentosum* L.), strawberry clover (*Trifolium fragiferum* L.), Talish clover (*Trifolium tumens* L.) and Caucasian clover (*Trifolium ambiguum* L.), predominantly in Australia, has focused on short-term tolerance morphological changes, nitrogen fixation and nodule morphology (Gibberd and Cocks, 1997; Stevenson et al., 2017; Enkhbat et al., 2021; Enkhbat et al., 2022).

Under field conditions and when exposed to water deficit, red clovers superior production and tolerance compared to white clover has been well documented (Peterson et al., 1992; Brock et al., 2003; Brown et al., 2005). There are few published controlled glasshouse water deficit experiments for red clover but Vaseva et al. (2011) identified stress tolerance related proteins that protect plant cells from destruction and improved their tolerance to stress conditions. Red clover was shown to significantly reduce shoot dry matter, relative water content and leaf area under prolonged water deficiency (Vaseva et al., 2011; Loucks et al., 2018). This has been proved to hamper recovery and reduce plant survival (Loucks et al., 2018). The objective of this study was to explore the physiological and phenotypic coping mechanisms of red clover (*Trifolium pratense L.*) germplasm populations under both water deficit and waterlogging. With the aim to (1) assess plant tolerance to water stress (water deficit and waterlogging) amongst a panel of sampled red clover germplasm accessions, (2) identify the coping mechanisms amongst accessions, and (3) identify the key morphological and physiological traits responsible for performance under water stress.

## 2 Methods

### 2.1 Plant material

A total of five red clover germplasm accessions (F3474, F4079, F3514, F2507 & F3594), one elite pre-breeding red clover top-cross line (JB4), one commercial red clover cultivar (Grasslands Relish) and one white clover cultivar (Grasslands Quartz) used in this experiment were sourced from the Margot Forde Germplasm Centre (MFGC) (**Table 1**). The five germplasm accessions were selected based on their place of origin and information on their collection sites. An accession is a distinct collection of uniquely identified seed, which is maintained as a part of a germplasm collection (National Academies of Sciences, Engineering, and Medicine, 1991). The elite line JB4 was selected due to its genetic background, it was produced from a top-cross involving plants from the cultivar Colenso and elite germplasm accessions. An elite line is defined as a group of plants with similar traits that are well adapted to environmental conditions in a targeted region. Which will be used for further breeding or released as a cultivar (National Academies of Sciences, Engineering, and Medicine, 1991; Falk, 2010). Relish and Quartz were selected as superior cultivar comparators, the latter added for interspecific comparison. Cultivars are a collection of plants with distinguishable characters created by breeders to meet specific breeding objectives (National Academies of Sciences, Engineering, and Medicine, 1991; Bernardo, 2002). From here onwards all accessions, elite lines and cultivars will be referred to as populations. A population is a collection of plants with similar backgrounds, individuals within potentially interbreed with one another (National Academies of Sciences, Engineering, and Medicine, 1991).

**Table 1:** Characteristics of the five red clover germplasm accessions, pre-breeding population and commercial cultivars.

Population	Accession	Country	City	Latitude	Altitude (m)	Annual rainfall (mm)	Collection site information
**1**	F3474	Azerbaijan	Quba	41.2°N	802	400-600	High rainfall flat meadow in the mountains by a river being cut for hay.
**2**	F4079	Greece	Lepida	41.4°N	1475	991	Forest with free draining alluvium soil.
**3**	F3514	Portugal	Monchique	37.5°N	84	499	Very rocky area with good drainage.
**4**	F2507	Spain	Viveiro	43.6°N	32	1102	Steep paddock near the sea.
**5**	F3594	Spain	Vilalba	43.2°N	424	1391	Low fertility area of lime-based track.
**6**	JB4	New Zealand	N/A	N/A	N/A	N/A	Elite pre-breeding population: maternal Colenso cultivar crossed with elite germplasm ecotypes.
**7**	Grasslands Relish	New Zealand	N/A	N/A	N/A	N/A	Commercial cultivar with a complex genetic background involving ecotypes from Spain, Portugal, Georgia, Azerbaijan, Yugoslavia, and New Zealand cultivars.
**8**	Grasslands Quartz	New Zealand	N/A	N/A	N/A	N/A	Commercial cultivar with a complex genetic background of elite cultivar populations of Saracen, Trophy and Tribute, with persistent germplasm ecotypes selected in New Zealand.

Information obtained from MFGC database, Ford and Barrett (2011), and Jahufer et al. (2021). N/A = information not available.

### 2.2 Experimental design

A randomized complete block design comprised of five replicates and three treatments containing 120 plants was used for this experiment. Each pot contained one plant. Six-liter plastic pots were filled with a soil mixture made with 75:25 ratio of Templeton silt loam soil and mortar sand. Additional nutrients were added for plant health and soil structure (**Supplementary Information**). This mixture is free draining to allow for the least resistant root growth and simpler separation of roots at final harvest (Ballizany et al., 2012). A further 108 potted red clover plants were placed next to the outer rows of pots, as a buffer, to minimized border effects. Plants were established in root trainers for 63 days and then transplanted into the pots and allowed to establish for 30 days before experiment initiation. The experiment ran for 52 days from treatment initiation between January 16th, 2021, and March 8th, 2021, at the AgResearch research farm glasshouse. The mean temperature was 24.4°C, average relative humidity 66% and daily solar radiation 19.5 MJ/m2.

### 2.3 Water stress treatments

All plants were watered to 15% volumetric soil moisture content (VMC) for 30 days to establish the experiment before initiation. Throughout the experiment the control plants maintained this status through watering by hand. In the water deficit stress treatment, plants were kept 3-4% above the wilting point of 5% VMC. This was measured gravimetrically, and the calculated amount of water was applied every 2-3 days (Hofmann et al., 2003). Plant populations were exposed to a minimum of 30 days water deficit stress. Waterlogging stress was applied by submerging the roots in a double pot system. The water level was kept at 2-3cm above pot height by encompassing the pot in a bucket filled with water. This system stopped the drainage of excess water, and the pots were topped up with water to ensure adequate submergence when needed (Simova-Stoilova et al., 2012). VMC was 15%, 5% and 50% for the control, water deficit, and waterlogged treatments, respectively. Plants were supported with bamboo poles when needed, prostrate growing accessions were not staked up to avoid false height measurements. The red clover populations were exposed to waterlogging for the first five days of the experiment between January 16^th^- 20^th^ 2021. When plants showed signs of stress through wilting the waterlogging treatment was stopped, and plants were allowed to recover. The waterlogging treatment for these plants was then reintroduced four days before the experiment finished. The white clover cultivar Quartz remained submerged for 52 days, as it showed no signs of wilting stress during that period.

### 2.4 Measurements

#### 2.4.1 Whole plant morphology and yield measurements

Plant height was measured on day 52 using a ruler at the highest point of the plant. Petiole length was measured on day 52 using a ruler on two randomly chosen young, fully unfolded leaves per plant. Dry matter percentage of shoots excluding leaves was calculated after the removal of leaves on day 52. Dry matter percentage was calculated by taking the ratio of dry matter over fresh matter then multiplied by 100. A fresh weight of the petiole and runner biomass was taken on day 52 prior to being oven dried at 80 °C for 48 hours, a dry weight was taken on removal. Estimated total dry matter was calculated per plant using the following equation,


$$\left(\left(TLFW*\left(\frac{LDW}{LWW}\right)\right)+RSDW\right)$$

where, TLFW is the total combined leaf fresh weight, LDW is the dry weight of two randomly chosen young, fully unfolded leaves, LWW is the fresh weight of the same two leaves and RSDW is the dry weight of the runners and petioles. An actual leaf dry weight was not possible due to the leaves being used for other destructive measurements. The total root dry mass was measured after the removal of the shoot biomass. Soil was carefully removed from the root dry mass using a sieve and water the day after shoot biomass was harvested. Once separated, roots were weighed once they were dried in an oven at 80°C for 48 hours.

#### 2.4.2 Leaf morphology

Two randomly chosen young, fully unfolded leaves per plant were harvested on day 52 and their lamina area was determined using the open-source image processing and analysis program ImageJ (Katabuchi, 2015). Subsequently, the lamina dry weight of these leaves was determined by being oven dried at 80°C for 48 hours. Relative leaf thickness was calculated using the following equation,


$${\left(SLA*RWC\right)}^{-1}$$

where, SLA is the specific leaf area and RWC is relative water content. SLA is calculated by the ratio of lamina area and lamina dry weight of two randomly chosen young, fully unfolded leaves per plant (Vile et al., 2005). Leaf fresh weight per plant was measured on day 52 by separating and weighing all the leaves from each plant. Average leaf size per plant was measured on day 52 using a qualitative visual 1-5 score (1: small, 2: small-medium, 3: medium, 4: medium-large, 5: large).

#### 2.4.3 Physiology/water status

Relative chlorophyll content was measured using a Konica Minolta SPAD-502Plus chlorophyll meter on day 52. An average reading was taken from three randomly chosen young, fully unfolded leaves per plant (Ling et al., 2010; Belachew and Stoddard, 2017). Relative water content was measured using the following equation,


$$RWC\left(\%\right)=100\;\left(\frac{FM-DM}{TM-DM}\right)$$

where, FM is the lamina fresh mass of two randomly chosen young, fully unfolded leaves per plant. TM is the lamina turgid mass after saturation of the two leaves in water for 24 hours and DM is the lamina dry mass after the leaves are oven-dried at 80°C for 48 hours (Marshall et al., 2001).

To measure osmotic potential, leaf samples were collected and frozen in 1.5ml Eppendorf tubes on day 52. Samples were then frozen in liquid nitrogen and centrifuged at 12,200 g for 5 minutes to separate the leaf sap. The sap was then pipetted onto filter paper discs and processed using a Wescor Vapro vapor pressure osmometer (model 5520). Prior to measurements the osmometer was calibrated using 0.1, 0.29, and 1 mmol/kg-1 osmolality standards. Regular cleaning between every 4 samples maintained the accuracy of the osmometer. The osmolality reading (mmol/kg^-1^) was used to calculate solute potential (*Ψ*π) using the following equation,


π=−RTcj

where, RT = 0.002437 m3 MPa·mol^-1^ at 20°C, and cj is the total solute concentration or osmolality (mmol·kg^-1^) (Cyriac et al., 2018).

### 2.5 Data analysis

Statistical analysis was carried out using the statistical software package Genstat 18th edition (VSN International, 2021) The unbalanced analysis of variance (ANOVA) using the regression model statistical method was selected for all trait analysis due to the incomplete block effect from plants dying during the experiment. After accounting for block effects, the ANOVA examined the levels of population and of water levels (control, water deficit and waterlogging) as the two main treatment factors, followed by the examination of the interaction of these two main factors. Raw trait data was either square root or log transformed to correct skewed data to conform to normality. Fisher’s unprotected least significant difference (LSD) test was used to separate treatment means wherever the ANOVA showed a significant treatment or interaction effect (P< 0.05). Interspecies differences were obtained using the comparisons contrast function within ANOVA in Genstat. Pattern analysis, a combination of cluster analysis and principal component analysis (PCA), was conducted using means generated from ANOVA, for traits with significant (P< 0.05) differences among populations using DeltaGen (Jahufer and Luo, 2018).

Prior to pattern analysis the data were standardized to remove scaling effects. The optimum number of entry groups from cluster analysis was determined according to Cooper and DeLacy (1994). The resulting biplot is a graphical summary of population by trait associations. Trait associations are presented through directional vectors, a positive relationship between traits is shown if vectors are separated by less than 90°. The results of the clustering analysis are superimposed on the biplot through assigning each group of populations a different color. Quartz was not included in the cluster analysis for water deficit and waterlogging to not affect the differences among the red clover populations. F2507 was not included in the cluster analysis for waterlogging as it did not survive the waterlogging treatment. Means and standard errors produced from ANOVA were used to generate column graphs.

## 3 Results

### 3.1 Population trait expression

The average trait expression of each of the eight populations across water treatments is summarized in **Table 2**. Both F3594 and Relish were classified as large leaved, tall erect populations with large root dry mass and at the high end of the scale for the other morphological traits. Both JB4 and F3474 averaged medium measurements for most of the traits. JB4 had a tall erect growth habit similar to F3594 and Relish but smaller root dry mass, leaf thickness, and leaf size. Populations F3514, F2507, and F4079 all measured as the smallest or lowest for all of the traits. These Populations were characterized as being small-leaved, with low biomass and prostrate growth. While trait expressions changed within each population across water treatments (**Tables 3**, **4**), there were no changes among populations. This indicated that the relative performance of populations that expressed the smallest or lowest for each trait under control conditions did not change under both water stress treatments.

**Table 2:** Heat map of average trait expressions amongst the eight populations averaged over all water treatments generated from an ANOVA analysis, 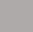 = small/low, 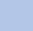 = medium, 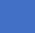 = large/high.

Trait Description	Pop1 F3474	Pop 2 F4079	Pop 3 F3514	Pop 4 F2507	Pop 5 F3594	Pop 6 JB4	Pop 7 Relish	Pop 8 Quartz
Total Dry Matter (g) **								
Runner Dry Matter (%) **								
Leaf Size (1-5) **								
Leaf Thickness (mm) **								
Plant Height (cm)***								
Petiole Length (cm) **								
Root dry mass (g)***								
Leaf Number **								
Root to Shoot Ratio (%) ***								
Relative Water Content (%) **								
Chlorophyll Content *								

Significance levels of main effect (*P< 0.05; **P< 0.01; *** P< 0.001)

**Table 3:** Significant changes for water deficit traits as compared to the control of each population (%), 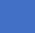 = increase, 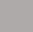 = decrease, □ = non-significant; Significance levels of main effect (*P< 0.05; **P< 0.01; *** P< 0.001).

Trait Description	Pop 1 F3474	Pop 2 F4079	Pop 3 F3514	Pop 4 F2507	Pop 5 F3594	Pop 6 JB4	Pop 7 Relish	Pop 8 Quartz	Average Red Clover	Average White Clover
Total Dry Matter (g) **	-30	-34	-48	-41	-43	-47	-40	-44		
Runner Dry Matter (%) **	12	9	7	10	6	9	6	16		
Leaf Size (1-5) **		-17		-33		-11		-23	-10	-29
Leaf Thickness (mm) **	-36	-41	-45	-39	-53	-54	-54	-64		
Plant Height (cm) ***	-12	-27		-29			-11	-27	-11	-43
Petiole Length (cm) **	-29	-40		-44		-25	-16	-31		
Root dry mass (g) ***					-27			-30		
Leaf Number **		-41	-58	-41	-58	-58	-48	-57		
Root to Shoot Ratio (%) ***	62	23	65	32	25	58	52			
Relative Water Content (%) **							-18	-20	-7	-22
Chlorophyll Content *					4			4		

Average red and white clover results derived from all respective populations under water deficit treatment using the comparison contrast function within the ANOVA function in Genstat.

**Table 4:** Significant changes for waterlogged traits as compared to the control of each population (%), 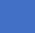 = increase, 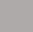 = decrease, □ = non-significant, X = plants did not survive Significance levels of main effect (*P< 0.05; **P< 0.01; *** P< 0.001).

Trait Description	Pop 1 F3474	Pop 2 F4079	Pop 3 F3514	Pop 4 F2507	Pop 5 F3594	Pop 6 JB4	Pop 7 Relish	Pop 8 Quartz	Average Red Clover	Average White Clover
Total Dry Matter (g) **	-33	-40	-27	**X**	-42	-43	-35	-14	-39	-2
Runner Dry Matter (%) **				**X**		9		-7	1	-11
Leaf Size (1-5) **	14	14		**X**						
Leaf Thickness (mm)**		-38		**X**	-45	-54	-30		-37	-3
Plant Height (cm) ***	-18			**X**	-13		-15	18		
Petiole Length (cm) **				**X**	26				6	22
Root dry mass (g) ***	-65	-95	-78	**X**	-62	-85	-64		-83	1
Leaf Number **	-41	-54		**X**	-58	-67	-32		-50	13
Root to Shoot Ratio (%) ***			-34	**X**				26	-24	67
Relative Water Content (%) **				**X**					-1	-10
Chlorophyll Content *	-3	-7	-4	**X**	-7	-10	-4			

Average red and white clover results derived from all respective populations under waterlogged treatment using the comparison contrast function within the ANOVA function in Genstat.

### 3.2 Water stress effects

**Figure 1:**
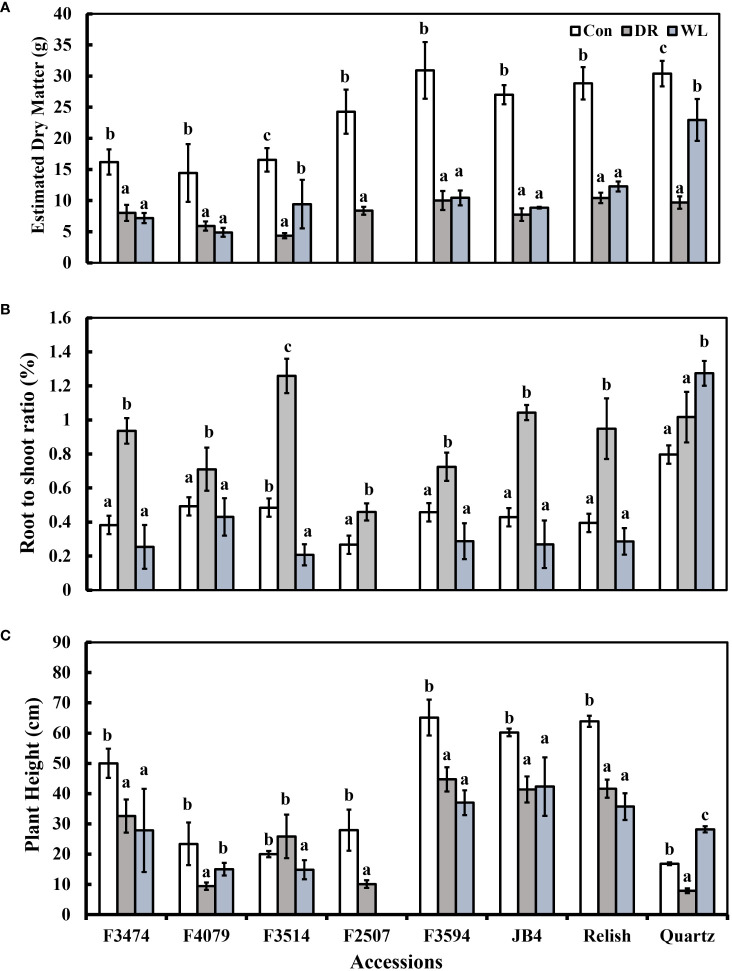
Plant response traits under control (Con), water deficit (DR) and waterlogging (WL) for the eight clover populations. **(A)** Total aboveground dry matter, **(B)** Root to shoot ratio and **(C)** Plant height. Statistical letters (a, b, c) indicate differences between treatment means within each population (P> 0.05; Fisher’s unprotected l.s.d. test, ± SE error bars, n = 5).

**Figure 2:**
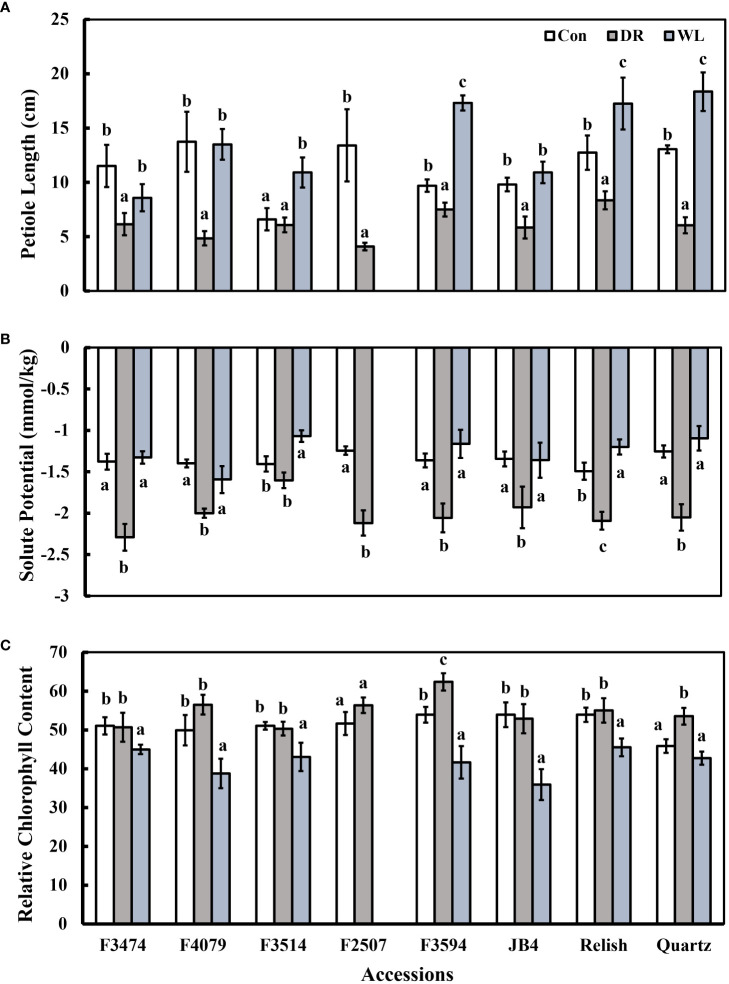
Plant response traits under control (Con), water deficit (DR) and waterlogging (WL) for the eight clover populations. **(A)** Leaf thickness, **(B)** Leaf size and **(C)** Leaf number. Statistical letters (a, ab, b, c) indicate differences between treatments within each population(P> 0.05; Fisher’s unprotected l.s.d. test, ± SE error bars, n = 5).

**Figure 3:**
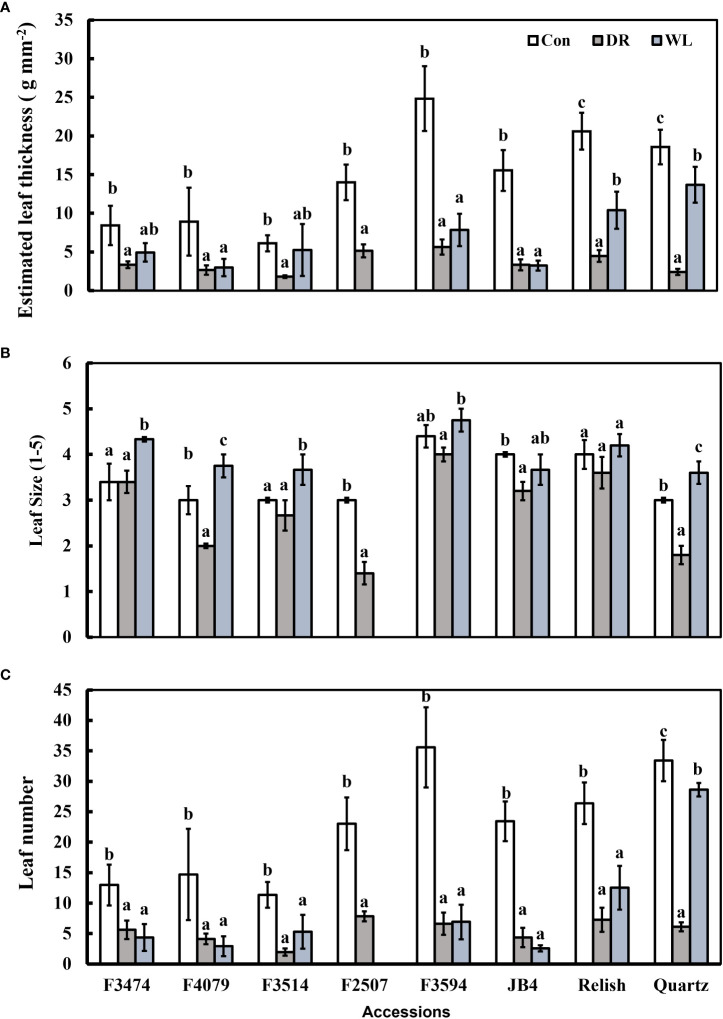
Plant response traits under control (Con), Water deficit (DR) and waterlogging (WL) for the eight clover populations. **(A)** Petiole length, **(B)** Solute potential and **(C)** Chlorophyll content. Statistical letters (a, b, c) indicate differences between treatments within each population (P> 0.05; Fisher’s unprotected l.s.d. test, ± SE error bars, n = 5).

Compared to control conditions, under water deficit and waterlogging, there were decreases in total dry matter (41% and 30%, respectively), root dry mass (19% and 30%, respectively), leaf thickness (50% and 28%, respectively), plant height (15% and 9%, respectively), and leaf number (50 and 34%, respectively); (**Figures 1A, C**, **2A, C**). Under waterlogging both petiole length (10%) and leaf size (8%) increased, in contrast under water deficit both decreased (25% for petiole length, 11% for leaf size) (**Figures 2A**, **3B**). Plant physiology and water status traits were affected by the different treatments, both chlorophyll content (5%) and solute potential (5%) increased under waterlogging but decreased under water deficit (2%, 23%, respectively) (**Figures 2B, C**). Under water deficit, both runner dry matter and root to shoot ratio increased (10%, 38%, respectively), relative water content also decreased by 10% (**Figure 1B**).

### 3.3 Water treatment x population interaction effects

Under water deficit, all populations significantly decreased total dry matter, but increased dry matter percentage (**Figure 1A**, **Table 3**). Plant height and petiole length significantly decreased for all populations except for F3514 and F3594, while JB4 also retained plant height (**Figures 1C**, **2A**). Both leaf thickness and leaf number were significantly decreased in all the populations except for F3474 which was able to maintain leaf number (**Figures 3A, C**). There was a significant decrease in leaf size for populations F4079, F2507, JB4 and Quartz (**Figure 3B**). Root dry mass decreased for populations F3594 and Quartz, while root to shoot ratio increased for populations F3474, F4079, F3514, F2507, F3594, JB4, and Relish (**Figure 1B**, **Table 3**). While relative water content significantly decreased for Relish and Quartz and chlorophyll content significantly increased for populations F3594 and Quartz (**Table 2**; **Figure 2C**).

Under waterlogging, total dry matter and root dry mass decreased for all the red clover populations while Quartz only had a significant reduction in total dry matter (**Figure 1A**). Leaf size increased for populations F3474 and F4079 (**Figure 3B**). Leaf thickness decreased in populations F4079, F3594, JB4 and Relish (**Figure 3A**). Plant height decreased for populations F3474, F3594 and Relish, while increasing for Quartz (**Figure 1C**). Petiole length increased significantly for population F3594 (**Figure 2A**). In all the red clover populations leaf number significantly decreased except for population F3514 (**Figure 3C**). Root to shoot ratio significantly decreased for population F3514 and significantly increased for Quartz (**Figure 1B**). In all the red clover populations chlorophyll content significantly decreased except for in population F3474 (**Figure 2C**).

### 3.4 Interspecific comparisons

Under water deficit, there were three significant differences between red clover and white clover responses. Leaf size and plant height decreased 10% and 11%, respectively for red clover and 29% and 43% for white clover, respectively. Relative water content was reduced 7% and 22% for red clover and white clover, respectively (**Table 3**). Under waterlogging there were large significant differences between red clover and white clover for total dry matter (39%, 2% decreases, respectively), root dry mass (83% decrease, 1% increase, respectively), leaf number (50% decrease, 13% increase, respectively), leaf thickness (37% and 3% decreases, respectively) and root to shoot ratio (24% decrease, 67% increase, respectively). There were lesser significant differences between red clover and white clover for dry matter percentage (1% increase, 11% decrease, respectively), petiole length (6% and 22% increases, respectively) and relative water content (1% and 10% decreases, respectively) (**Table 4**).

### 3.5 Water deficit plant trait response and cluster analysis

**Figure 4:**
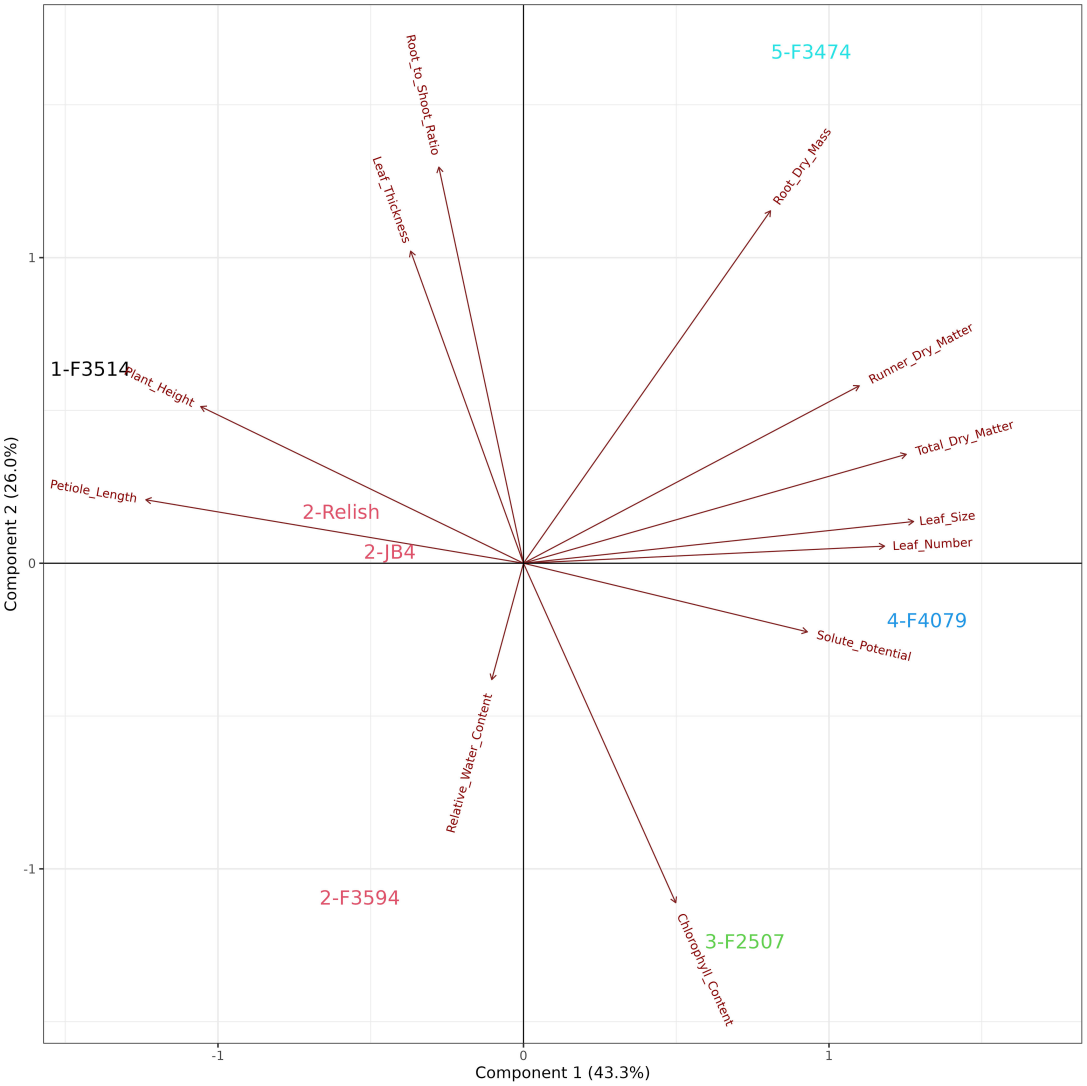
Biplot generated from principal component analysis of the red clover population by multi-trait best linear unbiased estimator (BLUE) matrix based on measurements from the water deficit treatment. The directional vectors indicate different traits. Population groups generated from cluster analysis are indicated using different colors (1-5).

The principal component analysis for water deficit response in the seven red clover populations showed that the first two principal components explained 69.3% of the variation in the data. The traits and their positive to negative associations with each other are indicated by the directional vectors (**Figure 4**). Differences between populations were derived from hierarchical cluster analysis. The seven red clover populations were clustered into five groups, indicated in color. Group 1; F3514 had large reductions in total biomass, leaf number and leaf thickness under water deficit. Group 2; F3594, JB4 and Relish had large reductions in leaf thickness and leaf number, along with a small change in leaf size, petiole length and plant height. Group 3; F2507 had high reductions in leaf size, plant height and petiole length. Group 4; F4079 had low reductions in total biomass, leaf thickness, leaf size and leaf number. It also had a large reduction in petiole length. Group 5; F3474 had lower reductions in total biomass, a high increase in dry matter percentage.

### 3.6 Waterlogging plant trait response and cluster analysis

**Figure 5:**
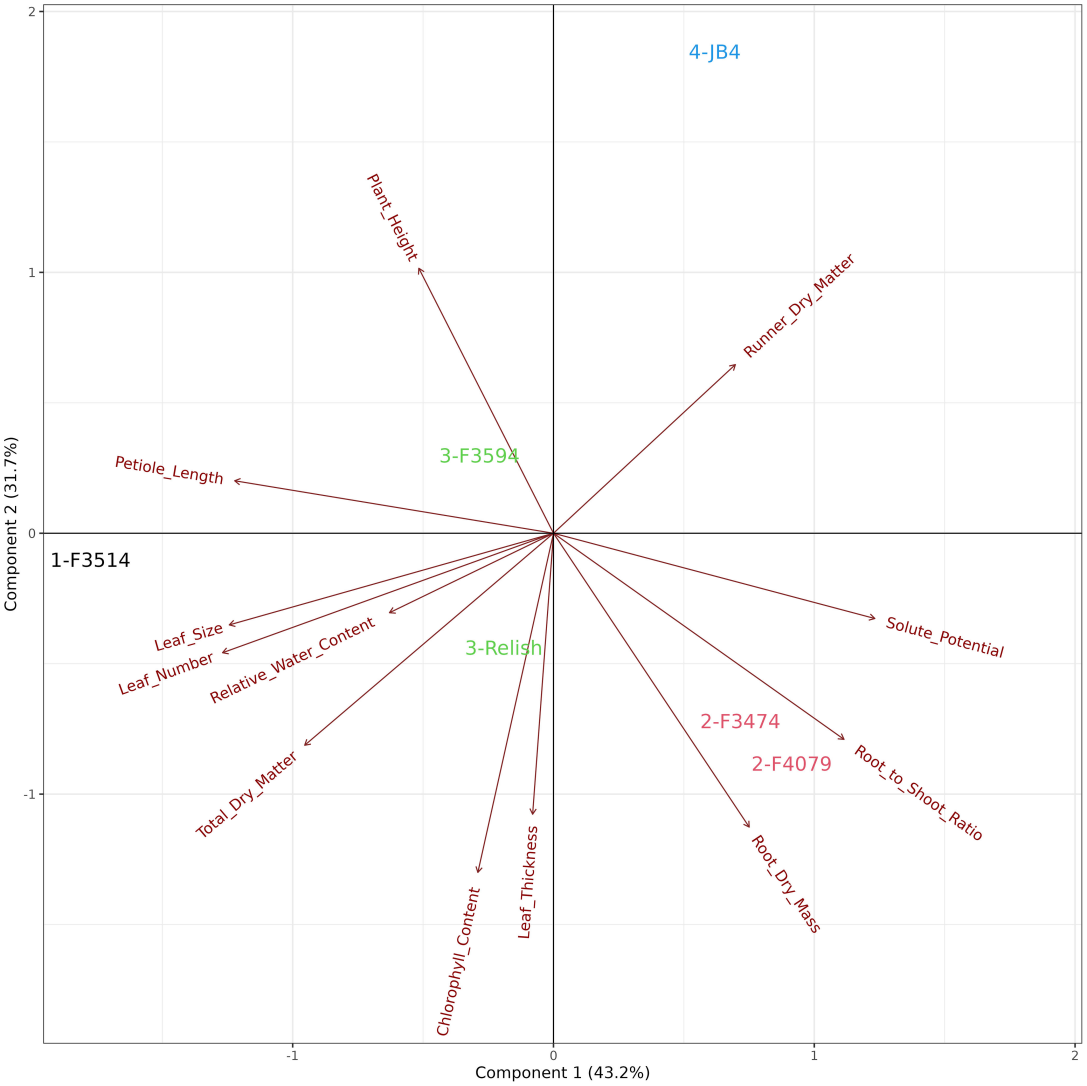
Biplot generated from principal component analysis of the red clover population by multi-trait best linear unbiased estimator (BLUE) matrix based on measurements from the waterlogging treatment. The directional vectors indicate different traits. Population groups generated from cluster analysis are indicated using different colors (1-4).

The principal component analysis for waterlogging response in six red clover populations showed that the first two principal components explained 74.9% of the variation in the data. The relationships among the traits are shown by the associations among the directional vectors color (**Figure 5**). Differences between populations were derived from hierarchical cluster analysis. The red clover populations were clustered into groups, indicated in Group 1; F3514 had a high reduction in root dry mass but was able to maintain similar measurements for the other traits. Group 2; F3474 and F4079 had high reductions in total biomass but had low reduction in root dry mass. Group 3; F3594 and Relish had low reductions in biomass, leaf thickness, leaf size and plant height. They also had a medium reduction in root dry mass. Group 4; JB4 had high reductions in total biomass, leaf thickness, leaf size and plant height.

### 3.7 Overall plant trait responses and cluster analysis

**Figure 6:**
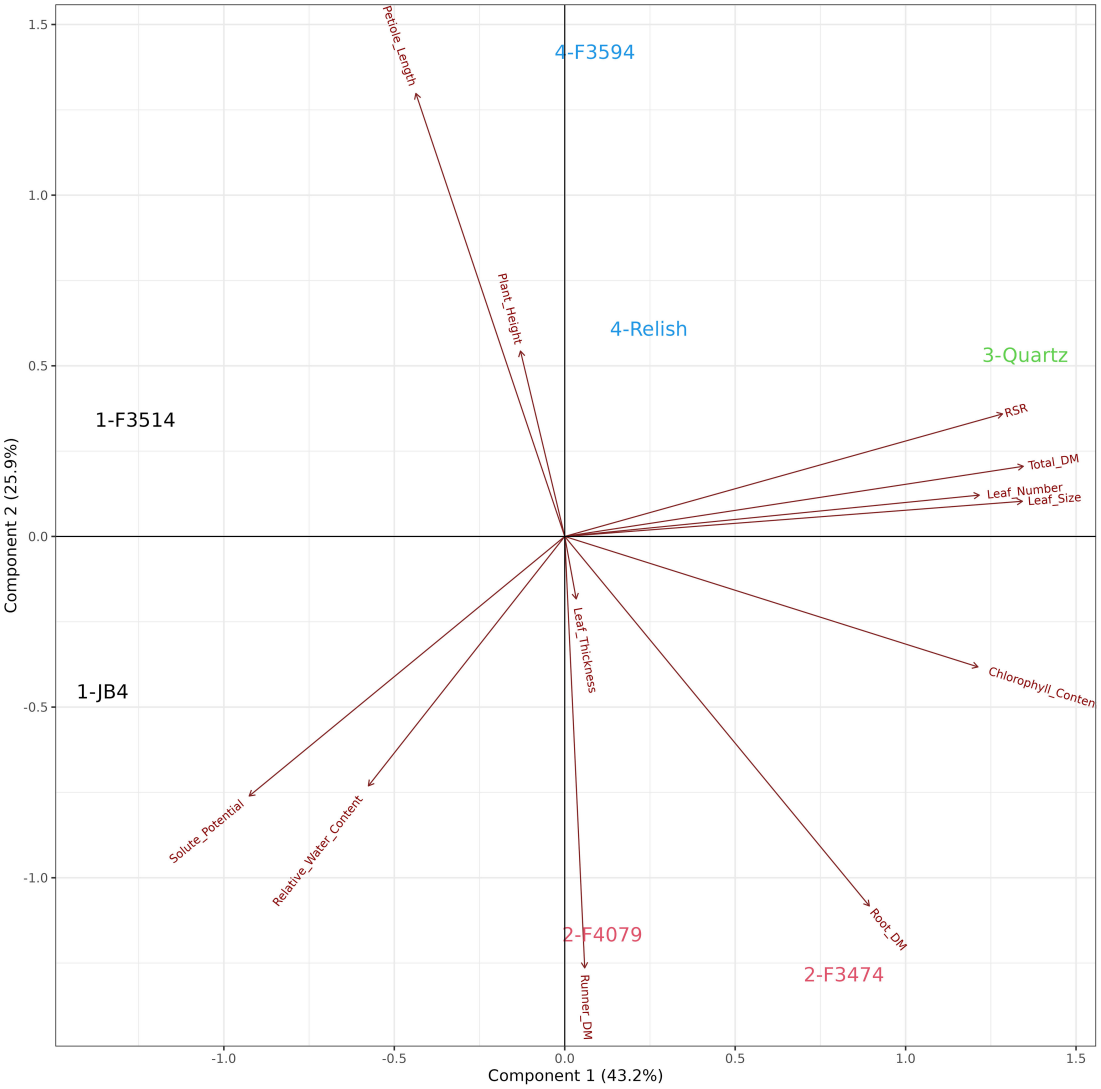
Biplot generated from principal component analysis of the red clover population by multi-trait best linear unbiased estimator (BLUE) matrix based on ratio changes across treatments. The directional vectors indicate different traits. Population groups generated from cluster analysis are indicated using different colors (1-4).

The principal component analysis for the combined waterlogging and water deficit stress plant response showed that the first two principal components explained 69.1% of the variation in the data (**Figure 6**). There were four groups of traits with multiple significant relationships with other traits. These groups can be identified as aboveground plant biomass traits (leaf number, total dry matter, chlorophyll content and leaf thickness), leaf response traits (petiole length and plant height), water status traits (relative water content and root to shoot ratio), and water deficit traits (runner dry matter percentage, root dry mass, leaf size and solute potential). The seven populations were clustered into groups, indicated in color which aligned with the four groups identified above (**Figure 6**). Group 1; F3514 and JB4 had high reductions in total biomass, root dry mass and number of leaves. Group 2; F4079 and F3474 had high reductions in plant height, petiole length and root dry mass. Group 3; Quartz had the lowest reductions in total biomass and number of leaves. It also increased chlorophyll content. Group 4; F3594 and Relish increased in petiole length and had medium reductions in total biomass. F2507 was not included in this analysis as it did not survive the waterlogging treatment.

## 4 Discussion

### 4.1 Whole plant morphology

The reduction of total dry matter, leaf number, petiole length and plant height under water deficit conditions is a consistent phenomenon highlighted in the literature (Peterson et al., 1992; White and Hodgson, 1999; Vaseva et al., 2011; Scoffoni et al., 2014). The severity of the reduction varies on individual plants as various plant organs respond differently (Wright and Westoby, 2002; Aroca, 2012). By reducing aboveground biomass under water deficit, root growth is prioritized over producing total dry matter. This provides adequate water for root growth from the limited supply until the plant can resume normal growth post water deficit. This results in an increase of root to shoot ratio. (White and Hodgson, 1999). All red clover populations prioritized maintaining root dry mass under water deficit by sacrificing shoot growth as evident by the increasing root-shoot ratio in our experiment. An increase in root to shoot ratio can be attributed to water deficit resistance, with active investment in root growth or maintenance the plant is able to access sufficient available water without fully depleting stored energy reserves (Aroca, 2012).

Under waterlogged conditions, the reduction of plant height, leaf number and total shoot dry matter has been found in red clover and other Trifolium species (Simova-Stoilova et al., 2012; Enkhbat et al., 2021). This reduction is an indication of the lack of tolerance to waterlogging (Bowley et al., 1984). In contrast, plants that tolerate waterlogging elongate shoot growth above the water level to maximize leaf area exposure, ensuring root aeration through the intake of oxygen and carbon fixation (Parent et al., 2008). Waterlogging tolerant species such as white clover are likely to increase root to shoot ratio due to the shift to the less efficient anaerobic energy production pathway needing more roots to support the same amount of growth (Striker, 2012a). Petiole length increased amongst waterlogged populations, this appeared to be the plants trying to efficiently intercept the most amount of light possible with fewer leaves by elongating the petiole above the water level (Black et al., 2009; Huber et al., 2009). Unlike the red clover populations, the white clover cultivar Quartz was able to elongate shoot growth above the water level and increase root to shoot ratio to support similar growth under the less efficient anaerobic energy production pathway.

For plants, developing organs such as leaves is a large investment and construction costs for each varies amongst species. The lifespan of these organs must allow the plant to cover initial investment costs and gain additional energy to promote plant growth (Westoby et al., 2002). When exposed to water stress how a plant invests the available resources changes, including what to invest into and at what scale. Other factors such as water level, light level and temperature can influence this investment. Leaf thickness and size decreased in water deficit plants, reflecting results shown in other clover studies (Huber et al., 2009; Ballizany et al., 2012; Scoffoni et al., 2014). Maintaining leaf thickness and size comes at a great cost to plants. However, by switching to producing fewer smaller leaves requires less energy and reduces transpiration water loss. The trade-off towards thicker leaves is an increase in durability and longevity (Westoby et al., 2002). While decreased leaf size and thickness is advantageous under restricted water availability it does reduce the agronomic productivity of a plant (Aroca, 2012). As in previous red clover studies (Mommer and Visser, 2005; Huber et al., 2009), the waterlogged plants produced larger and thinner leaves. In ideal conditions, plants that produce thinner leaves can increase biomass quicker by producing new leaves at faster rate. Under waterlogging, plants produced thinner leaves, and in previous studies this has been related to reductions in transpiration and photosynthesis (Huber et al., 2009; Striker, 2012b).

### 4.2 Root morphology

Root structure plays an important role in the storage of energy, response to water stress and subsequent recovery. As expected, both treatments caused a reduction in root dry mass. Similar observations on root structure were made by Christie and Martin (1999) and Aroca (2012). The differences in root dry mass (**Table 4**) under waterlogging conditions suggest that the white clover root system is better suited for these conditions, as Quartz was the only population that maintained root dry mass. Following water stress, the amount of reserve carbohydrates depleted during this period impacts the longevity and recovery of a plant. During water stress, plants switch to a less efficient anaerobic energy production pathway. Carbohydrates are used to keep up with energy demands. These are limited and so must be used efficiently and effectively to maintain growth. Plants that use less carbohydrate reserve or can continue to add to reserves during this period are able to resume pre-stress growth with more speed and vigor. Plants with very depleted reserves recover more slowly post-stress (Striker, 2012a). It can be suggested that under waterlogged conditions the red clover populations depleted their root reserves quickly resulting in slower recovery, whereas Quartz was able to continue growth close to pre-stress levels. The decreases in root dry mass (**Table 3**) under water deficit conditions suggest that the depletion of root reserves varied between populations.

### 4.3 Physiology/water status traits

Under drought conditions, relative water content decreases due to the reduced availability of water, resulting in a reduction of leaf size. A result of this is an increase in chlorophyll concentration (Ling et al., 2010). Chlorophyll content was measured here as an indicator for photosynthetic capacity of the populations, and there was a slight increase for two of the red clover water deficit populations. F3594, unlike the other red clover populations was able to actively raise chlorophyll content, indicating the photosynthetic capacity of these plants had increased (**Figure 3C**) (Palta, 1990; Ling et al., 2010). Solute potential also decreased amongst populations in response to the water deficit,

Under waterlogging, a reduction in chlorophyll content among the red clover populations indicated a decrease in photosynthetic activity, reducing three key morphological traits, total dry matter, leaf number and root dry mass. This showed a lack of tolerance to waterlogging as the populations spent stored energy but were not able to produce sufficient shoot growth. There is limited literature on both traits in red clover under waterlogging conditions, but similar results have been found in other leguminous species (Simova-Stoilova et al., 2012; Stevenson et al., 2017 ppr; Enkhbat et al., 2021; Kyu et al., 2021). Under waterlogging both traits are affected by the plants reduced uptake of oxygen and carbon dioxide due to stomatal closure and a decrease in the uptake of water and key nutrients such as nitrogen due to reduced root function (Striker, 2012b).

### 4.4 Overall population performance across water stress treatments

When developing cultivars, assessment of persistence and performance across multiple environments and stresses is crucial. This enables the identification of specific or broad adaptation amongst populations, and stress coping strategies. To our knowledge this is the first published research observing red clover germplasm populations under both water deficit and waterlogging treatments in the same controlled conditions. While no population exceeded expectation or showed full acclimatization under both treatments, four coping mechanisms were identified (**Figure 6**). Both F3514 and JB4 had high reductions in aboveground and root dry mass resulting in low performance across treatments and lack of broad adaptation. F2507 was not included in this analysis due to perishing under waterlogging after 5 days. The steep coastal land with high rainfall and good drainage conditions where F2507 originates from appears to have led to a lack of adaptation to waterlogging. Therefore, the lack of drainage under the waterlogging treatment appeared to be the key contributor to its death.

The remaining four red clover populations were categorized into two groups, large leaved erect populations, and small leaf prostrate populations. Both groups produced similar total dry matter using different isohydric (“saver”) or anisohydric (“spender”) coping mechanisms. The larger aboveground stature of F3594 and Relish required more energy to maintain than what was produced under stress. This significantly reduced the ability to maintain root dry mass. Whereas the smaller stature F4079 and F3474 populations reduced petiole length and plant height to maintain a high root dry mass. The performance of Quartz white clover appeared to be inflated by its superior performance under waterlogging, as it did not tolerate water deficiency well, with severe reductions in all morphological traits but similar total dry matter to the other populations. Overall, Quartz was able to maintain a larger total biomass than the red clover populations, but further research is recommended to validate its adaptive ability over both stresses. The fact that Relish did not outperform the other germplasm populations under waterlogging, identifies an area to target in future breeding programs.

This study identified key morphological and physiological responses to water stress extremes amongst red clover populations, highlighting the different coping mechanisms applied by differing morphological stature populations under water deficiency and waterlogging. Root dry mass was identified across both water treatments as a key trait, a plant’s ability to maintain root dry mass was a key contributor to its overall growth and survival. The size and number of key aboveground morphological traits such leaf number, leaf size, petiole length and plant height influenced how a plant responded to the water stress. Better understanding the effects of water stress on key plant traits will enable breeders to select important ones in breeding programs. The introduction of raw germplasm for evaluation reveals new genetics for selection and will provide the basis the development of stress-adapted cultivars.

## Data availability statement

The raw data supporting the conclusions of this article will be made available by the authors, without undue reservation.

## Author contributions

AH, ZJ and RH designed the experiment and contributed to data analyses and the writing of the manuscript. AH performed the experiment and analysed the data. All authors contributed to the article and approved the submitted version.
