# Supplementary material for: Responses to water stress extremes in diverse red clover germplasm accessions
Source: Front Plant Sci. 2023 Jun 22;14:1195058. doi: 10.3389/fpls.2023.1195058 (PMC10325626; doi:10.3389/fpls.2023.1195058)
Supplement: Supplementary file 1 [file DataSheet_1.pdf]

Supplementary Material

Trait responses to water stress extremes in diverse red clover germplasm accessions

Angus D. Heslop [1,2*], Zulfi Jahufer[3], Rainer W. Hofmann[1*]

*** Co-correspondence:**
Angus Heslop
angus.heslop@agresearch.co.nz

Rainer Hofmann
rainer.hofmann@lincoln.ac.nz

1.1 Soil mix for pots

The potting mix for this experiment is a mixture of Wakanui silt loom, sand and fertiliser. This soil mixture allows the plant to establish roots throughout the pot and ensures a fine, crumbly soil that washes of the roots well at harvest preserving roots for an accurate harvest (Ballizany, 2011).

1) 75 % Wakanui silt loam

2) 25 % mortar sand 0-3 mm

3) 2 g / l Osmocote Exact Standard 3-4 months., N-P-K 16-5.0-9.2 + 1.8 mg trace elements +

1.8% Magnesium, 16% Nitrogen Total (N), 7.1% Nitrate nitrogen (NO3-N), 8.9% Ammoniacal nitrogen (NH4-N), 5% Phosphorus Pentoxide (P2O5), 5% Water soluble, 9.2% Potassium oxide (K2O), 9.2% Water soluble, 1.8% Magnesium oxide (MgO), 1.3% Water soluble, 0.40% Iron (Fe), 0.20% Chelated by EDTA, 0.06% Manganese (Mn), 0.02% Boron (B), 0.02% Water soluble, 0.047% Copper (Cu), 0.03% Water soluble, 0.02% Molybdenum (Mo), 0.014% Water soluble, 0.015% Zinc (Zn)

4) 1 g / l Hydraflo wetting agent

5) 30 g / l gypsum (calcium sulphate, CaSO4)

1.2 ANOVA tables for the twelve morphological and physiological traits

Petiole length	d.f.	s.s.	m.s.	v.r.	F pr.
Block	4	0.30	0.08		
Line	7	3.80	0.54	6.73	<.001
Treatment	2	12.16	6.08	75.33	<.001
Line.Treatment	14	3.11	0.22	2.76	0.002
Residual	76	6.13	0.08		
Total	103	25.51	0.25		

Plant height	d.f.	s.s.	m.s.	v.r.	F pr.
Block	4	0.70	0.17		
Line	7	30.71	4.39	46.08	<.001
Treatment	2	5.86	2.93	30.78	<.001
Line.Treatment	14	6.86	0.49	5.15	<.001
Residual	77	7.33	0.10		
Total	104	51.46	0.49		

Runner dry matter	d.f.	s.s.	m.s.	v.r.	F pr.
Block	4	0.15	0.04		
Line	7	0.64	0.09	3.8	0.001
Treatment	2	2.46	1.23	51.24	<.001
Line.Treatment	13	0.76	0.06	2.45	0.008
Residual	76	1.82	0.02		
Total	102	5.82	0.06		

Total dry matter	d.f.	s.s.	m.s.	v.r.	F pr.
Block	4	2.75	0.69		
Line	7	26.93	3.85	11.68	<.001
Treatment	2	79.09	39.55	120	<.001
Line.Treatment	13	9.31	0.72	2.17	0.019
Residual	74	24.39	0.33		
Total	100	142.47	1.42		

Root to shoot ratio	d.f.	s.s.	m.s.	v.r.	F pr.
Block	4	0.07	0.02		
Line	7	1.23	0.18	9.28	<.001
Treatment	2	1.83	0.92	48.22	<.001
Line.Treatment	13	0.94	0.07	3.8	<.001
Residual	72	1.37	0.02		
Total	98	5.45	0.06		

Root dry mass	d.f.	s.s.	m.s.	v.r.	F pr.
Block	4	2.17	0.54		
Line	7	26.15	3.74	13.69	<.001
Treatment	2	23.26	11.63	42.62	<.001
Line.Treatment	13	14.87	1.14	4.19	<.001
Residual	75	20.46	0.27		
Total	101	86.91	0.86		

Number of leaves	d.f.	s.s.	m.s.	v.r.	F pr.
Block	4	2.72	0.68		
Line	7	50.34	7.19	7.99	<.001
Treatment	2	107.37	53.68	59.63	<.001
Line.Treatment	13	28.94	2.23	2.47	0.007
Residual	74	66.62	0.90		
Total	100	255.98	2.56		

Leaf size	d.f.	s.s.	m.s.	v.r.	F pr.
Block	4	0.17	0.04		
Line	7	3.80	0.54	23.39	<.001
Treatment	2	1.99	0.99	42.91	<.001
Line.Treatment	13	0.88	0.07	2.93	0.002
Residual	77	1.79	0.02		
Total	103	8.62	0.08		

Leaf thickness	d.f.	s.s.	m.s.	v.r.	F pr.
Block	4	0.27	0.07		
Line	7	26.89	3.84	8.22	<.001
Treatment	2	65.63	32.82	70.21	<.001
Line.Treatment	13	14.86	1.14	2.45	0.008
Residual	74	34.59	0.47		
Total	100	142.24	1.42		

Relative water content	d.f.	s.s.	m.s.	v.r.	F pr.
Block	4	3.02	0.76		
Line	7	9.56	1.37	4.72	<.001
Treatment	2	9.06	4.53	15.66	<.001
Line.Treatment	14	7.66	0.55	1.89	0.04
Residual	76	21.98	0.29		
Total	103	51.28	0.50		

Chlorophyll content	d.f.	s.s.	m.s.	v.r.	F pr.
Block	4	0.36	0.09		
Line	7	0.19	0.03	2.42	0.027
Treatment	2	1.08	0.54	48.03	<.001
Line.Treatment	13	0.32	0.02	2.18	0.018
Residual	77	0.87	0.01		
Total	103	2.81	0.03		

Solute potential	d.f.	s.s.	m.s.	v.r.	F pr.
Block	4	0.23	0.06		
Line	7	0.21	0.03	3.06	0.007
Treatment	2	1.76	0.88	88.19	<.001
Line.Treatment	13	0.22	0.02	1.66	0.087
Residual	75	0.75	0.01		
Total	101	3.16	0.03		
